# Microglial replacement in the aged brain restricts neuroinflammation following intracerebral hemorrhage

**DOI:** 10.1038/s41419-021-04424-x

**Published:** 2022-01-10

**Authors:** Xiuping Li, Xiaolin Gao, Wenyan Zhang, Mingming Liu, Zhaoli Han, Minshu Li, Ping Lei, Qiang Liu

**Affiliations:** 1 Department of Neurology, Aging and Neurodegenerative Disease Laboratory, Tianjin Neurological Institute, Tianjin Medical University General Hospital, Tianjin, China; 2 Department of Neurology, Shenzhen Institute of Translational Medicine, The First Affiliated Hospital of Shenzhen University, Shenzhen Second People’s Hospital, Shenzhen, China; 3 Department of Geriatrics, Tianjin Geriatrics Institute, Tianjin Medical University General Hospital, Tianjin, China

**Keywords:** Neuroimmunology, Cognitive ageing

## Abstract

Aged microglia display augmented inflammatory activity after neural injury. Although aging is a risk factor for poor outcome after brain insults, the precise impact of aging-related alterations in microglia on neural injury remains poorly understood. Microglia can be eliminated via pharmacological inhibition of the colony–stimulating factor 1 receptor (CSF1R). Upon withdrawal of CSF1R inhibitors, microglia rapidly repopulate the entire brain, leading to replacement of the microglial compartment. In this study, we investigated the impact of microglial replacement in the aged brain on neural injury using a mouse model of intracerebral hemorrhage (ICH) induced by collagenase injection. We found that replacement of microglia in the aged brain reduced neurological deficits and brain edema after ICH. Microglial replacement-induced attenuation of ICH injury was accompanied with alleviated blood-brain barrier disruption and leukocyte infiltration. Notably, newly repopulated microglia had reduced expression of IL-1β, TNF-α and CD86, and upregulation of CD206 in response to ICH. Our findings suggest that replacement of microglia in the aged brain restricts neuroinflammation and brain injury following ICH.

## Introduction

Inflammation is a critical aggravator of neural injury following brain insults. In the aged brain, microglia exhibit an exaggerated and uncontrolled inflammatory phenotype in response to brain insults or immune stimulation [1]. Rather than an enhanced immune response at baseline level, aged microglia possess a primed profile that is demonstrated by augmented production of inflammatory factors such as interleukin (IL)−1β and reactive oxygen species following stimulus [2]. Although evidence suggests a link between the primed profile of the aged microglia and vulnerability of the old brain to inflammation-related secondary injury following acute insult [3], it remains poorly understood to what extent the aged microglia with a primed profile can impact the neuroinflammation and the outcome of acute brain injury.

The survival of microglia critically depends on signaling through the colony‐stimulating factor 1 receptor (CSF1R) [4]. Administration of CSF1R inhibitor PLX3397 eliminates microglia in the whole brain that continues when CSF1R inhibition is present [4]. Moreover, removal of CSF1R inhibition stimulates the rapid repopulation of the entire brain with new microglial cells [4, 5], leading to effective replacement of the entire microglia population, a process takes approximately 2–3 weeks to complete. Recent evidence suggests that withdrawal of CSF1R inhibitors in the old mice leads to complete repopulation of new microglia with characteristics resembling young microglia. Therefore, withdrawal of CSF1R inhibitors in the old brain resets the primed microglia and provides an opportunity to determine the impact of aged microglia on neural injury upon brain insults [6]. In this study, we investigated the impact of microglial replacement in the aged brain on neural injury using a mouse model of intracerebral hemorrhage (ICH) induced by collagenase injection.

## Materials and methods

### Animals

All animal experiments were conducted in accordance with the National Institutes of Health Guide for the Care and Use of Laboratory Animals in China approved by the Committee on the Ethics of Animal Experiments of Tianjin Neurologic Institute. The study complies with the ARRIVE (Animal Research: Reporting in vivo Experiments) guidelines. Male C57BL/6 mice (16–20 months) were purchased from the Vital River Corporation (Beijing,China). All animals were housed in pathogen-free conditions with free access to food and water.

### Experimental design

**Fig. 1:**
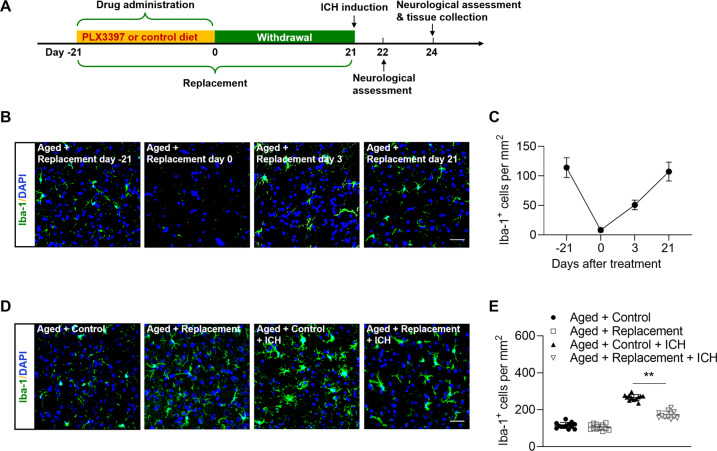
Microglial replacement in the aged brain. Groups of aged mice received 21 days of PLX3397 treatment in chow or a control diet, and then followed by three weeks of withdrawal prior to ICH induction. ICH induction, neurological assessment and tissue collection were conducted at indicated time points. **A** Flow chart illustrates drug administration and experimental design. **B** Brain sections from groups of aged mice receiving PLX3397 in chow at indicated time points after treatment or withdrawal were stained with Iba-1 (green). Nuclei were stained with DAPI (blue). Scale bar: 50 µm. **C** Counts of Iba-1^+^ cells in indicated groups of mice. *n* = 8 per group. **D** Brain sections from groups of aged mice receiving indicated treatments were stained with Iba-1 (green). Nuclei were stained with DAPI (blue). Scale bar: 50 µm. **E** Counts of Iba-1^+^ cells in indicated groups of mice. *n* = 12 per group. Data are presented as mean ± SD. **p < 0.01.

Male C57BL/6 mice were used in this study. Mice were randomly assigned to following experimental groups: (1) Aged + Control (*n* = 20); (2) Aged + Replacement (*n* = 36); (3) Aged + Control + ICH (*n* = 72), (4) Aged + Replacement + ICH (*n* = 72). The timeline of experiments (drug administration, ICH induction, neurological assessment and tissue collection) was illustrated in Fig. 1A.

### Compound and drug administration

PLX3397 (Selleckchem Inc, Houston, TX) was formulated in a standard chow (1022, Beijing HFK Bioscience Co. Ltd., China). Groups of mice received PLX3397 (290 mg/kg of chow) for 21 days to eliminate microglia as previously described [4, 7], and then followed by withdrawal for 21 days prior to ICH induction. Mice in control groups received the standard chow (1022, Beijing HFK Bioscience Co. Ltd., China).

### Induction of ICH in mice

ICH was induced by injection of bacterial collagenase in mice as previously described [8–10]. Mice were fixed on a stereotactic frame after anesthesia. Mice were anesthetized with an intraperitoneal injection of a ketamine (100 mg/kg) and xylazine (10 mg/kg) mixture. After a midline scalp incision, 0.0375 U bacterial collagenase (Type IV-S, Sigma) was injected to the right basal ganglia (coordinates 0.5 mm anterior and 2.5 mm lateral to the bregma and at a depth of 3.7 mm beneath the surface of the skull). Collagenase was dissolved in 0.5 μl saline and injected at a rate of 0.5 μl /min using a microinfusion pump. Following infusion, the needle was removed after a 20 min pause. The burr hole was filled with bone wax, and mice were placed in cages with enough food and water. During the surgery, mouse body temperature was maintained by a electrical blanket to 37 ± 0.5 °C.

### Neurologic assessment

Neurologic deficits were assessed at days 1 and 3 after ICH by at least 2 investigators blinded to treatment. The modified Neurologic Severity Score (mNSS), corner-turning test, and foot-fault test were performed as described [8–10]. The mNSS rates neurologic functioning on a scale of 15 and includes a composite of motor, sensory, reflex, and balance tests. For corner-turning test, the mouse was allowed to proceed into a corner with an angle of 30° and was required to turn either to the left or the right to exit the corner. Foot-fault test was used to assess a rodent’s sensorimotor function. Mice were placed in a grid device measuring 32 cm × 20 cm × 50 cm (length × width × height) with a mesh size of 12 mm and allowed to roam freely for 5 min. A foot fault was defined as the limb dropping into the grid hole or resting with the grid at the wrist level. The percentage of foot faults was calculated as the following formula: foot faults / (foot fault + non foot fault steps) × 100.

### Brain water content

Brain water content was measured on day 3 after ICH as previously described [8–10]. The mice were anesthetized by intraperitoneal injection, then brain tissues were obtained and divided into 3 portions: the ipsilateral hemisphere, the contralateral hemisphere, and cerebellum. The collected tissues were weighed immediately to obtain the wet weight, followed by drying for 24 h at 100 °C to measure the dry weight. The calculation formula of brain water contents was: (wet weight – dry weight)/wet weight × 100%.

### Flow cytometry

Flow cytometry was used to detect the counts of peripheral immune cells and cytokine production in brain and spleen as previously described [9–11]. At day 3 after ICH and perfusion with PBS, mice brain tissues were collected and digested with collagenase IV to form a single cell suspension. After removing myelin using 30% Percoll solution, cell pellets were harvested on the bottom of the tube and suspended in 1% BSA solution for staining. To harvest splenic cells, the spleen tissues were removed and mechanically homogenized through 70 um nylon cell strainers in PBS on ice. Cell pellets were prepared for staining after lysis of red blood cells. All antibodies were purchased from Biolegend (San Diego, CA, USA), unless otherwise indicated. The following antibodies were used in this study: CD45 (30-F11), CD11b (M1/70), Ly6G (1A8), Ly6C (HK1.4), CD3 (145-2C11), CD4 (GK1.4), CD8 (53-6.7), NK1.1 (PK136), CD19 (1D3), CD86 (GL-1), CD206 (C068C2), tumor necrosis factor alpha (TNF-α) (MP6-XT22), interlukin-6 (MP5-20F3). IL-1β (NJTEN3) was ordered from eBioscience (SanDiego, CA, USA). Flow cytometry was conducted on FACS Aria flow cytometer and data were analyzed by Flow Jo 7.6.1 software.

### Immunostaining

Mice were euthanized and perfused with PBS followed by 4% paraformaldehyde (PFA), and then whole brain removed, fixed in PFA followed by 15% and 30% sucrose to dehydrate. The brains were embedded in OCT for preparation of frozen sections. Brain sections (5 μm thick) were incubated in permeabilizing and blocking solution (5% donkey serum, 3% BSA, and 0.3% Triton 100 in PBS solution) for 1 h at room temperature. The sections were incubated with primary antibodies at 4 °C overnight. The following primary antibodies were used: Iba-1(019-19741; Wako), CD31 (AF3628; R&D Systems), Claudin-5 (35–2500; Invitrogen), ZO-1 (33-9100; Invitrogen), NeuN (ab177487; Abcam), and caspase-3 (43-7800; Invitrogen). After washing with PBS, slices were incubated with appropriate fluorochrome conjugated secondary antibodies: donkey anti-rabbit 488 (Invitrogen, Carlsbad, CA), donkey anti-mouse 546 (Invitrogen, Carlsbad, CA), donkey anti-goat 488 (Invitrogen, Carlsbad, CA) respectively, at room temperature for 1 h. Finally, all the slices were incubated with fluoroshield mounting medium with DAPI (Abcam, Cambridge, MA). Images were captured by fluorescence microscopy (Olympus, model BX-61). Image analysis was performed using Image J software.

### Statistical analysis

All values are presented as mean ± SD. Statistical details are provided in the figure legends. No statistical methods were used to predetermine sample sizes, but our sample sizes are similar to those reported in previous publications [7–11]. Animals were randomly assigned to experimental groups. For animal studies, no data points were excluded. Data distribution was assumed to be normal, but this was not formally tested. All results were analyzed by investigators blinded to experimental groups using GraphPad Prism 7 Software (GraphPad Inc.). Two-tailed unpaired Student’s *t* test was performed for determining the significance of differences between two groups. Two-way ANOVA accompanied by Bonferroni post hoc test was used for multiple comparisons. Statistical significance was set at *p* < 0.05.

## Results

### Replacement of microglia in the aged brain

To replace microglia in the aged brain, we fed aged mice with a CSF1R inhibitor PLX3397 in chow or a control diet for three weeks. Thereafter, these mice were subjected to withdrawal of PLX3397 for additional three weeks, and then followed by ICH induction by collagenase injection. Prior to ICH induction, we measured the counts of microglia in the brains from groups of ICH mice receiving PLX3397 in chow versus a control diet. We found that three weeks of repopulation allows the counts of microglia to recover to baseline level (Fig. 1A–C). Following PLX3397 treatment and repopulation, we found that the count of microglia following ICH was reduced in the aged mice as compared to aged ICH mice receiving the control diet (Fig. 1D, E), suggesting reduced activity of microglia in the aged brain following replacement.

### Reduced ICH injury in the aged brain following microglial replacement

**Fig. 2:**
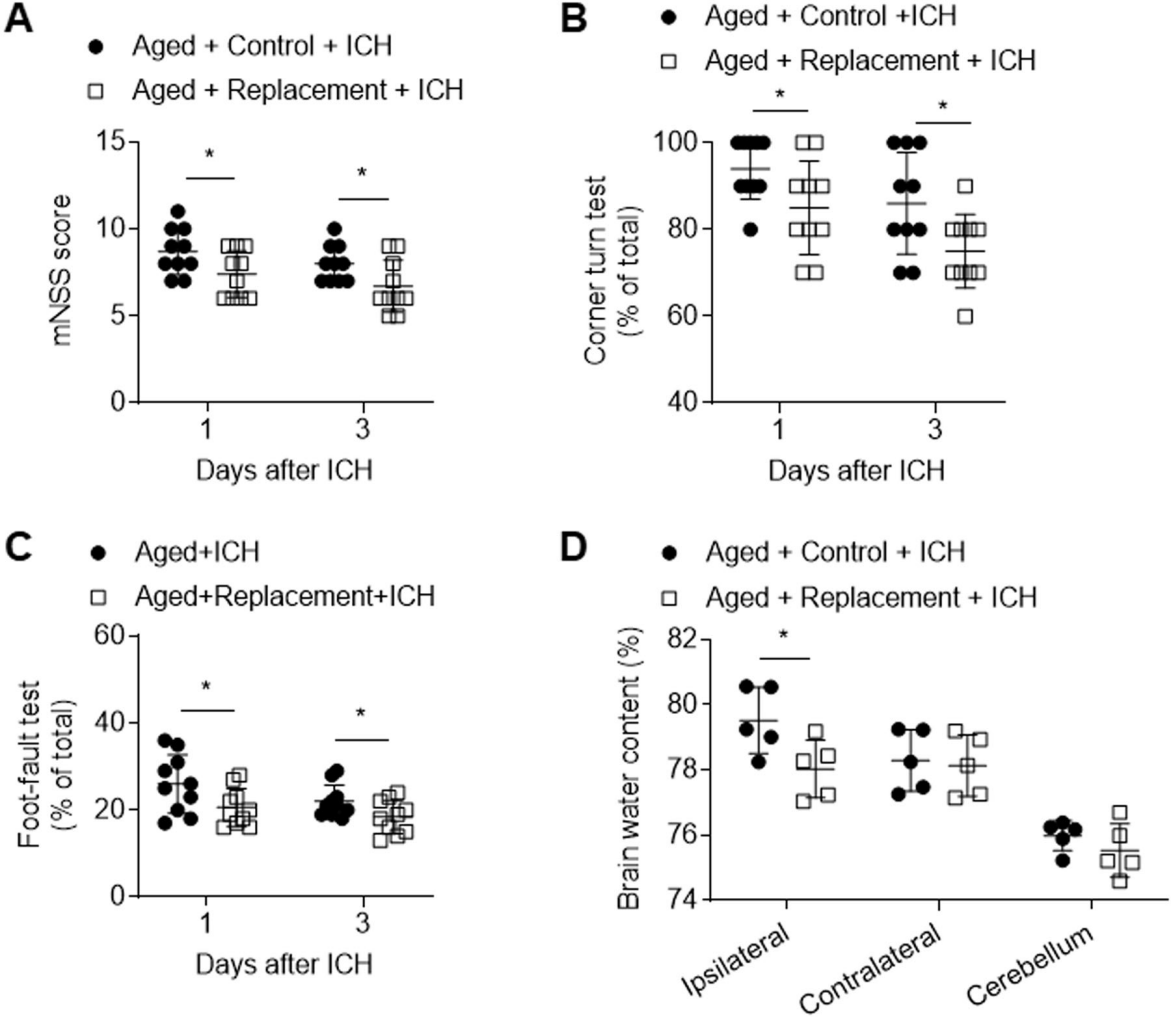
Effects of microglial replacement on neurological deficits and brain water content in the aged mice following ICH. ICH was induced in mice by injection of collagenase. **A**–**C** Summarized results showing neurological assessment (mNSS score, corner-turning test, and foot-fault test) of indicated groups of mice. *n* = 10 per group. **D** Brain water content in groups of mice receiving indicated treatments at day 3 after ICH. *n* = 5 per group. Data are presented as mean ± SD. *p < 0.05.

Microglia initiate neuroinflammation and contribute to brain edema following ICH [12, 13]. To assess the potential impact of microglial replacement on ICH injury in the aged mice, we measured neurological deficits and brain water content in the aged mice receiving PLX3397 in chow or a control diet followed by three weeks of withdrawal prior to ICH induction. Interestingly, in the aged mice receiving microglial replacement, we found reduced neurological deficits and brain water content following ICH (Fig. 2A–D).

### Microglial replacement in aged brain attenuated blood-brain barrier disruption and neuronal death after ICH

**Fig. 3:**
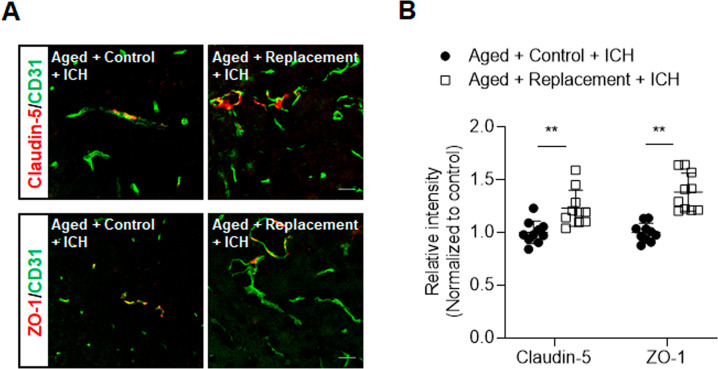
Effects of microglial replacement on blood-brain barrier integrity after ICH in the aged mice. ICH was induced in mice by injection of collagenase. **A** Brain sections from ICH mice in microglial repopulation group or control group were stained with CD31 (green), Claudin-5 (red), and ZO-1 (red) at day 3 after ICH. Scale bar: 20 µm. **B** Summarized results show that microglial repopulation had reduced Claudin-5 and ZO-1 loss in immunofluorescence intensity within the lesion area after ICH in aged mice. *n* = 10 sections from two mice per group. Data are presented as mean ± SD. **p < 0.01.

**Fig. 4:**
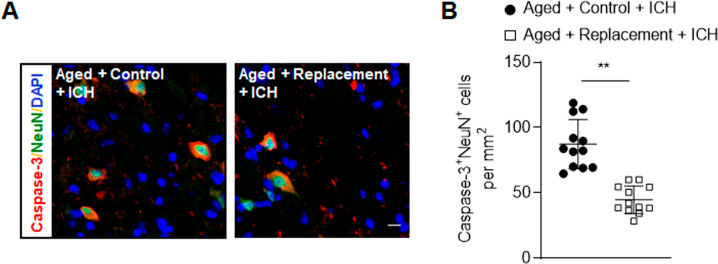
Effects of microglial replacement on neuronal death after ICH in the aged mice. ICH was induced in mice by injection of collagenase. **A** Brain sections from groups of ICH mice receiving indicated treatments after ICH were stained with NeuN (green) and Caspase-3 (red) at day 3 after ICH. Nuclei were stained with DAPI (blue). Scale bar: 10 µm. **B** Counts of Caspase-3^+^NeuN^+^ cells from groups of ICH mice receiving indicated treatments after ICH. *n* = 12 per group. Data are presented as mean ± SD. **p < 0.01.

As blood-brain barrier disruption is a significant contributor to brain edema after ICH, we determined the effects of microglial replacement on blood–brain barrier integrity in the aged brain after ICH. Immunostaining of ZO-1 and claudin-5 at 72 h after ICH revealed that microglial replacement preserved tight junction proteins (Fig. 3A, B). In addition, microglial replacement in the aged brain significantly reduced the counts of NeuN^+^Caspase-3^+^ cells in the perihematomal area (Fig. 4A, B), suggesting that microglial replacement reduces neuronal death following ICH.

### Microglial replacement suppresses leukocyte infiltration and microglia activity in the aged brain following ICH

**Fig. 5:**
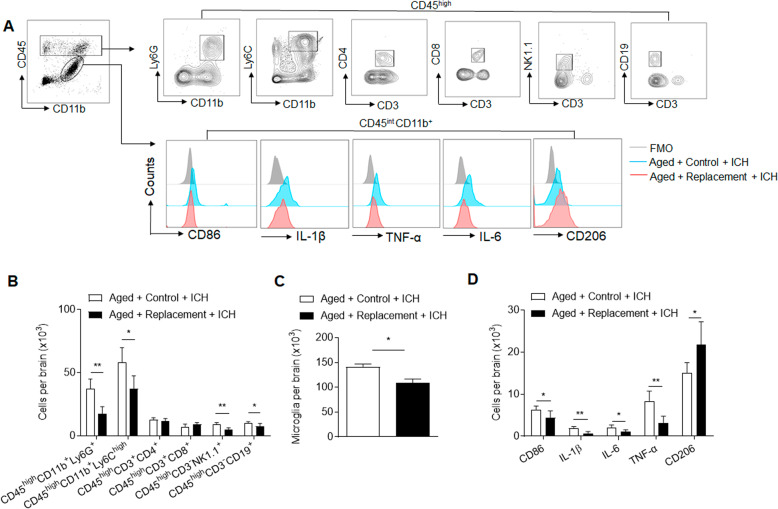
Effects of microglial replacement on leukocyte infiltration and microglia activity in the aged brain following ICH. ICH was induced in mice by injection of collagenase. At day 3 after ICH, brain tissues were obtained for flow cytometry analysis. **A** Gating strategy of brain-infiltrating neutrophils (CD45^high^CD11b^+^Ly6G^+^), monocytes (CD45^high^CD11b^+^Ly6C^high^), NK cells (CD45^high^CD3^-^NK1.1^+^), CD4^+^ T cells (CD45^high^CD3^+^CD4^+^), CD8^+^ T cells (CD45^high^CD3^+^CD8^+^), and B cells (CD45^high^CD3^-^CD19^+^), as well as microglia (CD11b^+^CD45^int^) and markers of their inflammatory activity (CD86, TNF-α, IL-6, IL-1β, CD206). **B** Counts of brain-infiltrating leukocyte subsets in the brains from indicated groups of ICH mice. **C**, **D** Counts of microglia and their expression CD86, TNF-α, IL-6, IL-1β, and CD206 in the brains from indicated groups of aged mice after ICH. *n* = 5 mice per group. Data are presented as mean ± SD. *p < 0.05, **p < 0.01.

Next, we assessed the influence of microglial replacement on leukocyte infiltration into the ICH brain. Using flow cytometry, we measured the counts of immune cell subsets in the brain of aged ICH mice receiving microglial replacement (Fig. 5A). We found significantly reduced counts of microglia (CD11b^+^CD45^int^), neutrophils (CD45^high^CD11b^+^Ly6G^+^), monocytes (CD45^high^CD11b^+^Ly6C^high^), NK cells (CD45^high^CD3^-^NK1.1^+^), and B cells (CD45^high^CD3^-^CD19^+^) in the brain of aged ICH mice receiving microglial replacement versus control diet (Fig. 5B, C). In contrast, the counts of other immune cell subsets such as CD4^+^ T cells (CD45^high^CD3^+^CD4^+^) and CD8^+^ T cells (CD45^high^CD3^+^CD8^+^) remain unaltered in the brain of aged ICH mice receiving microglial replacement (Fig. 5B). Notably, microglial replacement reduced the counts of microglia expressing CD86, IL-1β, IL-6, and TNF-α (Fig. 5D). The counts of microglia expressing CD206 were increased (Fig. 5D). These results demonstrate that microglial replacement suppresses brain infiltration of leukocytes and inflammatory activity of microglia in the aged brain following ICH.

### Microglial replacement has no significant impact on splenocytes in the aged mice following ICH

**Fig. 6:**
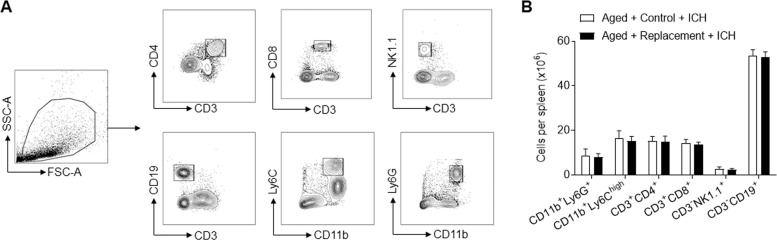
Effects of microglial replacement on splenocytes in the aged mice following ICH. ICH was induced in mice by injection of collagenase. At day 3 after ICH, spleen tissues were obtained for flow cytometry analysis. **A** Gating strategy of neutrophils (CD11b^+^Ly6G^+^), monocytes (CD11b^+^Ly6C^high^), NK cells (CD3^-^NK1.1^+^), CD4^+^ T cells (CD3^+^CD4^+^), CD8^+^ T cells (CD3^+^CD8^+^), and B cells (CD3^-^CD19^+^). **B** Cell counts of indicated immune cell subsets in spleens of aged mice after ICH. *n* = 5 per group. Data are presented as mean ± SD.

We also investigated the impact of microglial replacement on immune subsets in the spleens from aged mice. Results from flow cytometry analysis revealed similar counts of neutrophils (CD11b^+^Ly6G^+^), monocytes (CD11b^+^Ly6C^high^), NK cells (CD3^-^NK1.1^+^), CD4^+^ T cells (CD3^+^CD4^+^), CD8^+^ T cells (CD3^+^CD8^+^), and B cells (CD3^-^CD19^+^) in the spleens from aged mice following ICH (Fig. 6). These results suggest that microglial replacement in the aged brain has no significant impact on splenocytes in the aged mice following ICH.

## Discussion

In this study, we provide novel evidence that microglial replacement in the aged brain exerts beneficial effects against ICH injury. As documented here, microglial depletion and repopulation in the aged brain led to reduced inflammatory activity of microglia, i.e., down-regulation of inflammatory markers, in response to ICH. We also found ameliorated blood-brain barrier disruption and leukocyte infiltration in the aged ICH brain from mice receiving microglia replacement. As a result, microglia replacement in the old brain led to alleviated neurological deficits, brain edema, and neuronal death following ICH. During the aging process, microglia undergo significant changes in their morphology and function, including increased counts, dystrophic morphology, reduced phagocytosis and motility, and augmented responsiveness to environmental stimuli [14, 15]. These microglia in the old brain are often described as “primed” or “senescent”, leading to the escalated baseline of inflammation in the aged brain that is a key contributor to exacerbated neural injury following brain insults. As the survival of microglia requires signaling through CSF1R, CSF1R inhibition leads to a nearly complete depletion of microglia [7]. Following the withdrawal of CSF1R inhibition, new microglia repopulate the old brain within weeks and possess properties resembling microglia in the young brain [6]. Since microglia in the aged brain are known to worsen the local environment through the production of inflammatory factors such as reactive oxygen species and cytokines [16, 17], it is reasonable to postulate that the replacement of these “old” microglia in the aged brain with “young” microglia may lead to alleviation of neuroinflammation in the setting of ICH. Indeed, we found replacement of microglia in the aged brain reduces the inflammatory activity of microglia in response to ICH, leading to reduced barrier disruption and leukocyte infiltration. As a result, we found reduced neurological deficits and brain edema in the aged mice receiving microglia replacement, indicating its beneficial effects on ICH injury.

The major functions of microglia comprise of phagocytosis, producing inflammatory factors and antigen presentation, etc. Phagocytosis is a major feature of microglia. Following ICH, the upregulation of CD206 in the newly repopulated microglia suggests augmentation of endocytosis and phagocytosis. Accumulating evidence identifies alternatively activated microglia expressing CD206 as a pivotal contributor to hematoma clearance [3]. Therefore, these microglia may represent a beneficial aspect of microglial replacement that could both reduce the acute injury and facilitate functional recovery after ICH. The reduced expression of co-stimulation molecule CD86 and inflammatory cytokines such as IL-1β and TNF-α implies mitigated inflammatory activity in the old microglia after replacement. IL-1β and TNF-α are major inflammatory cytokines that exacerbate barrier disruption and neuronal death after ICH [3]. Therefore, the replacement of aged, primed microglia with “young” microglia could reduce brain inflammation after acute injury and improve neurological outcomes. In line with our findings, previous study also shows that microglial replacement in the old mouse brains improves the density dendritic spine and cognition [6]. Together with our finding that microglial replacement reduces neuronal death, these results support the notion that microglial replacement-induced resolution of neuroinflammation leads to reduced damage of neuronal structure in the old brain following acute insults. Recent studies also suggest an active role of vessel-associated microglia that are enriched near the vasculature to maintain blood-brain barrier integrity by the expression of tight-junction protein such as Claudin-5 and making physical contact with endothelial cells [18, 19]. In this regard, the replacement of aged microglia may lead to an increase of vessel-associated microglia that provide benefit to maintain blood-brain barrier integrity following ICH.

In this study, we used PLX3397 to eliminate microglia. In addition to numerous advantages of this pharmacological approach, the use of PLX3397 has limitations including potential off-target effects on tyrosine kinases c-Kit and Fms-like tyrosine kinase 3 [20]. Alternative genetic approaches have also been utilized to deplete microglia such as toxin-based models. However, these approaches only offer short-lived microglial depletion and induce a cytokine storm that require extra caution when interpreting findings using these models. Nevertheless, currently available techniques to deplete microglia have their own limitations regarding the extent and duration of depletion, off-target effects and other factors. As a result, caveats should be taken to correctly interpret the findings employing these approaches. Although our results assign a beneficial role of microglia replacement in the aged brain against acute ICH injury, still unclear is the long-term impact of microglial replacement on ICH prognosis. Further studies are needed to determine the long-term outcome and mechanisms underlying the potential effects of microglial replacement on brain recovery following ICH.

In summary, our study reveals that microglial replacement in the aged brain attenuates neuroinflammation and brain injury after ICH, suggesting that it may be a promising candidate for preclinical ICH studies.

## Supplementary information

Reproducibility checklist

## Data Availability

The datasets and other information that support the findings of this study are available from the corresponding author upon reasonable request.
